# Mutational heterogeneity in non-serous ovarian cancers

**DOI:** 10.1038/s41598-017-10432-9

**Published:** 2017-08-29

**Authors:** Jamie K. Teer, Sean Yoder, Anxhela Gjyshi, Santo V. Nicosia, Chaomei Zhang, Alvaro N. A. Monteiro

**Affiliations:** 1 Department of Biostatistics and Bioinformatics, H. Lee Moffitt Cancer Center and Research Institute, Tampa, FL 33612 USA; 2 Molecular Genomics Core Facility, H. Lee Moffitt Cancer Center and Research Institute, Tampa, FL 33612 USA; 3 Cancer Epidemiology Program, H. Lee Moffitt Cancer Center and Research Institute, Tampa, FL 33612 USA; 4 University of South Florida Cancer Biology PhD Program, Tampa, FL 33612 USA; 5 Department of Pathology and Cell Biology, USF Morsani School of Medicine, University of South Florida, Tampa, FL 33612 USA

## Abstract

Epithelial ovarian cancer is a leading cause of death in gynecological cancers. While several systematic studies have revealed the mutation landscape of serous epithelial ovarian cancer, other non-serous subtypes of the disease have not been explored as extensively. Here we conduct exome sequencing of nine non-serous epithelial ovarian tumors (six endometrioid and three mucinous) and their corresponding normal DNA as well as a tumor-only granulosa cell sample. We integrated the exome data with targeted gene sequencing for 1,321 genes selected for their involvement in cancer from additional 28 non-serous ovarian tumors and compared our results to TCGA ovarian serous cystadenocarcinoma and uterine corpus endometrial carcinomas. Prevalence of *TP53* mutations in non-serous was much lower than in serous epithelial OC, whereas the prevalence of *PIK3CA*, *PIK3R1, PTEN*, *CTNNB1*, *ARID1A*, and *KRAS* was higher. We confirmed the high prevalence of *FOXL2* and *KRAS* mutations in granulosa cell tumors and in mucinous tumors, respectively. We also identified *POLE* proofreading domain mutations in three endometrioid ovarian tumors. These results highlight mutational differences between serous and non-serous ovarian cancers, and further distinguish different non-serous subtypes.

## Introduction

Epithelial Ovarian Cancer (EOC) is a heterogeneous disease with five major histologic types^1, 2^: high-grade serous carcinoma, which accounts for the approximately 70% of all EOC cases, and clear cell (10%), endometrioid (10%), mucinous (3%), and low-grade serous carcinomas (<5%). These subtypes differ in terms of histopathology, morphology, and genomic alterations and are considered distinct diseases, which require careful diagnosis and specific therapies that are critical for successful treatment^3^. Ovarian granulosa cell tumors do not have epithelial origin but are the most common sex cord-stromal tumor and represent 5% of all ovarian malignancies^4^.

Based on molecular profiles, disease development and prognosis, the different histological subtypes can be hierarchically grouped into type I and type II^5^. Type I tumors are slow growing and encompass low-grade serous, low-grade endometrioid, mucinous and clear cell carcinomas characterized by mutations in *KRAS*, *BRAF*, and *PIK3CA* and by the absence of *TP53* mutations^6^. Type II tumors are highly aggressive and encompass high-grade serous tumors as a typical representative, as well as high-grade endometrioid and undifferentiated carcinomas with disruption of *TP53* ^7^. Type II tumors have expression profiles that cluster separately from type I tumors and are characterized by genomic instability, extremely aggressive clinical progression and poor prognosis^8–11^.

A comprehensive view of the germline variation associated with cancer risk^12–22^ and somatic alterations in high-grade serous ovarian carcinoma has recently emerged (Supplementary Table 1)^8, 23–25^. These tumors were characterized by somatic *TP53* mutations in 96% of all samples and low prevalence mutations in nine additional genes such as *NF1*, *BRCA1*, *BRCA2*, *RB1* and *CDK12*. Recurrent large copy number alterations were observed (8 gains and 22 losses) and included genes such as *MYC* and *KRAS* (gain) and *PTEN*, *RB1*, and *NF1* (loss). Many of these larger gains and losses were observed in the majority of tumors, highlighting the genomic instability of high-grade serous ovarian carcinoma. In addition, analyses of complete genomes for sixteen low stage high grade serous EOC revealed frequent *TP53* mutations, tetraploidy and homologous recombination repair defects^26^.

However, other non-serous forms of ovarian cancer contribute to significant morbidity and mortality yet few systematic studies have been conducted to characterize their germline and somatic mutational profile. Recently, genome-wide association studies identified three susceptibility loci and analysis of somatic alterations revealed a more detailed view of mucinous ovarian cancer^27, 28^. Most published studies, however, report germline or somatic targeted sequencing of a limited number of genes^9, 29–31^, or exome studies with a limited number of samples^32^ (Supplementary Table 1). A comparison between endometrioid uterine and endometrioid ovarian carcinomas using select exon capture of mutation profiles revealed that despite a possible common origin in endometriosis these tumors display differences in their mutation profile^33^.

In order to further explore the genomic landscape of non-serous ovarian cancer, we conducted exome sequencing of ten non-serous (endometrioid, mucinous, and granulosa) tumors and performed targeted gene sequencing of exons in 1,321 genes for 28 additional tumor samples including the clear-cell subtype. We report mutational and copy number profiles indicating that non-serous ovarian cancer is genomically distinct from serous EOC and displays similarities to non-serous endometrial uterine cancer.

## Results

Tumors were chosen by interrogating Moffitt tissue bank for cases that fulfilled the following criteria: a) non-serous histology; b) Stage I; and c) available fresh-frozen tissue; d) available matched normal blood or tissue. Whole exome sequencing was performed on 9 tumor/normal matched pairs, and one tumor only referred to as the ES (exome sequencing) cohort (Table 1). No case with clear cell tumor fulfilled the selection criteria. A median of 184 million reads and 17.5GB of exome sequencing data per sample was obtained. We annotated 32,965 somatic mutations within 100 bases of the exome target region (median = 1,299; range 829–15,767 per tumor, Supplementary Table 3) for an overall rate of mutations of 12.1 per Mb. The overview of the study is depicted in a REMARK (Reporting recommendations for tumor marker prognostic studies)-style diagram (Supplementary Figure 1).

**Table 1:** Samples used in exome sequencing.

Case	Histology	Grade	Stage	Age at diagnosis	Tissue of normal DNA	Main findings
OV1	Endometrioid Adenocarcinoma	FIGO 2	1C	48	Uterus	Glandular and sparse solid patterns. Focal squamous differentiation.
OV2	Endometrioid Adenocarcinoma	FIGO 2	1C	63	Blood	Glandular pattern.
OV3	Endometrioid Adenocarcinoma	FIGO 1	1A	28	Blood	Glandular and villoglandular patterns. Adenofibroma component.
OV4	Endometrioid Adenocarcinoma	FIGO 2	1C	49	Blood	Glandular and sparse solid patterns.
OV5	Endometrioid Adenocarcinoma	FIGO 1	1C	53	Blood	Glandular pattern. Focal squamous differentiation.
OV6	Endometrioid Adenocarcinoma	FIGO 1	1A	54	Blood	Glandular pattern.
OV7	Granulosa Cell tumor	Ungraded	1A	57	N/A	Adult type, trabecular pattern.
OV8	Mucinous Adenocarcinoma	NS	1A	54	Blood	Endocervical type. *BRCA2* germline pathogenic variant.
OV9	Mucinous Adenocarcinoma	NS	1A	41	Blood	Endocervical type.
OV10	Mucinous Adenocarcinoma	NS	1C	44	Blood	Endocervical type. LMP neoplasm component.

N/A, not available; NS, grading not standardized; LMP, low malignant potential.

### Common cancer exome alterations

**Figure 1:**
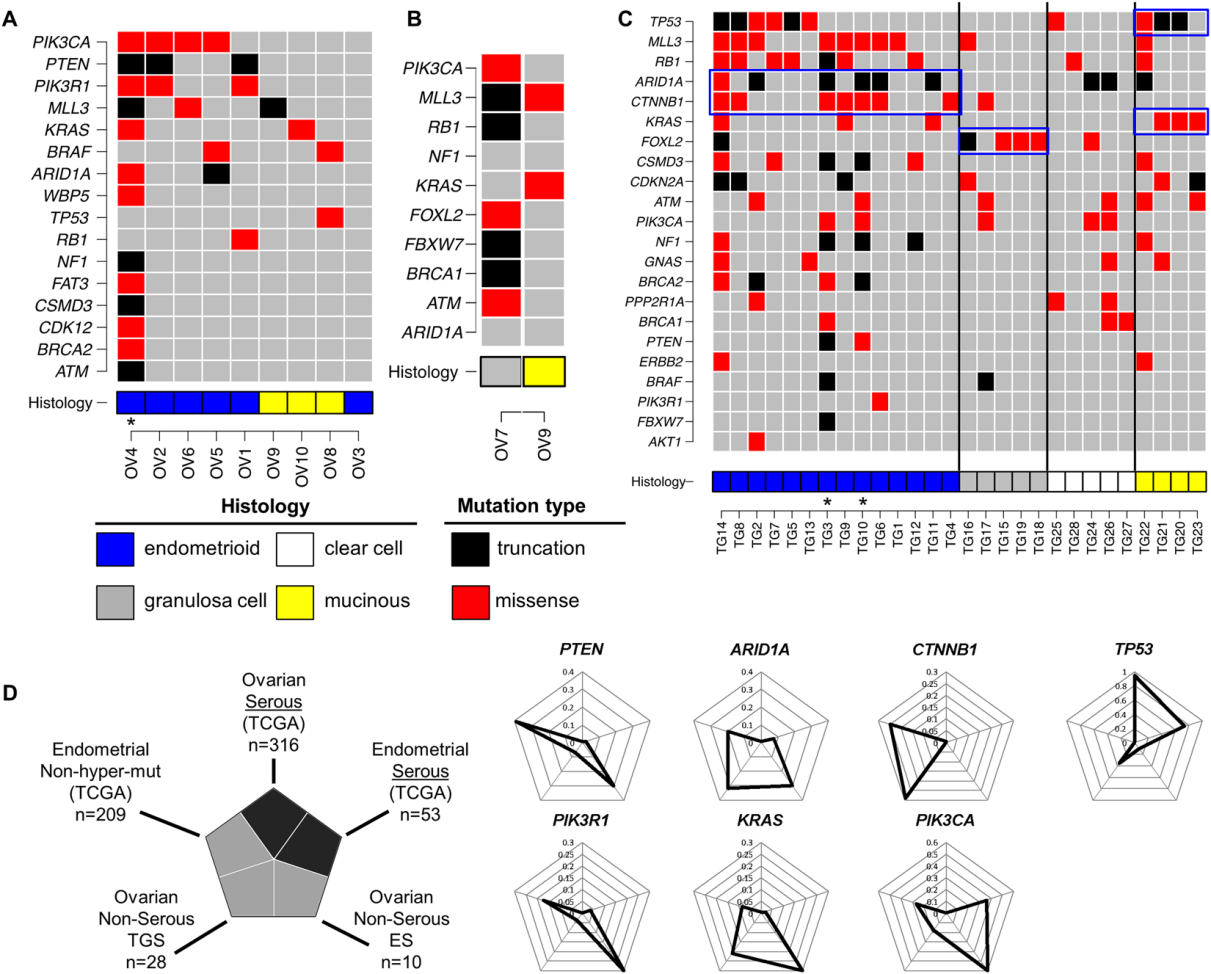
Mutation profile in non-serous ovarian cancer. (**A**) Mutation profiles showing mutations identified using tumor-normal pair analysis. Each column corresponds to exome sequencing data for one ovarian tumor. Rows correspond to presence or absence of mutation in the sample. (**B**) Mutation profiles showing mutations identified using tumor-only analysis. Each column corresponds to exome sequencing of one ovarian tumor. Rows correspond to presence or absence of mutation in the sample. (**C**) Mutation profiles showing mutations identified in target gene sequencing. Each column corresponds to one ovarian tumor. Rows correspond to presence or absence of mutation in the sample. Blue boxes highlight the high prevalence of specific mutations in certain histological subtypes. Sample histologies are shown in different colors representing endometrioid (blue), mucinous (yellow), clear cell (white), and granulosa cell (grey) histologies. Missense mutations and truncations are shown as red and black boxes, respectively. Star indicates samples with identified *POLE* proofreading domain mutations. (**D**) Frequency of the most common mutations in different tumor types and histologies. The radar graph display the frequency of various mutations across datasets, clockwise from top: ovarian serous cystadenocarcima (TCGA), serous endometrial uterine cancer (TCGA), non-serous ovarian tumors (exome sequencing, this study), non-serous ovarian tumors (target gene sequencing, this study), and non-hypermutable endometrial uterine cancers (TCGA). Height of the peak from the middle of the radar indicates the frequency of individuals with a mutation in the given gene.

MutSigCV_1.4 was used to identify significantly mutated genes, but no genes were observed with significant q-value. Only three genes were observed with q-value <1: *GOLGA6L1* (q = 0.62), *PTEN* (q = 0.73), and *OR2T33* (q = 0.76). No single confident mutated position (<1% in 1000 Genomes and 238 TCC normal samples, passing manual review) was observed in more than three samples, and only *PTEN* stood out as a gene containing truncating mutations in three samples (Fig. 1A). Mutations were mapped and compared to the COSMIC database (http://cancer.sanger.ac.uk/cosmic). Several COSMIC cancer genes were found to have various mutations in multiple samples, including *PTEN*, *PIK3CA*, *BRCA2*, and *ATM* (Fig. 1A, Supplementary Table 4). Several known cancer mutations were seen in unique samples (*KRAS* p.G12D, *BRAF* p.D594G, *NF1* p.R1362*, and *ZFAND1* p.R130*). Only one sample (OV8) had a *TP53* mutation (p.G245D, seen 22x in TCGA breast, ovarian, and colorectal cancers).

To ensure no mutations were missed due to local low coverage in the normal sample, we also identified genotypes using GATK on tumor samples alone. Mutations previously observed in COSMIC were retained. No further *TP53* mutation was observed. Additional cancer mutations were detected in OV7 (*FOXL2* p.C134W) and OV9 (*KRAS* p.G12V) (Fig. 1B). We observed a somatic p.Q257H variant in *BRAF* but the functional consequence of this alteration is unclear. Although *NRAS* was well covered (97.4% bases covered ≥10× on average) in our dataset, no mutations were observed, suggesting it may not be a common driver of mucinous tumors. Interestingly, for sample OV10, the *KRAS* variant was only called in the tumor/normal analysis (Fig. 1A) but not in tumor only analysis (Fig. 1B). The variant base was present in 4/23 reads in the tumor and 0/11 reads in the normal. We confirmed this variant is present in heterozygosis by Sanger sequencing in OV10.

GATK-based analysis of OV10 also detected a large insertion in *PIK3CA*, a missense variant in *ARID1A* (p.Ala532Thr) observed in only 2/25 tumor and 0/100 normal reads, and an *NF1* large insertion that introduces a stopgain. Sanger sequencing did not confirm those changes suggesting that they may have been incorrectly called or that only a small percentage of cells in the tumor carry that variant.

We also assessed germline variants in the exome sequencing data from the normal samples available from 9 cases (Supplementary Tables 9 and 10; except OV7) focusing on a set of genes implicated in ovarian cancer^34^. Only one clearly pathogenic variant, *BRCA2*:c.6944_6947delTAAA (p.Ile2315Lysfs) (OV8) variant and few variants of uncertain clinical significance were identified (Supplementary Table 10). We confirmed the *BRCA2* mutation using Sanger sequencing. A list of all non-synonymous variants called using a combination of Mutec and Strelka, including variants within the SeqCap EZ Exome v3 regions + 100 bp flanking sequence, is shown in Supplementary Table 11. We excluded variants observed in 1000 Genomes Project (Phase 3) or in an internal dataset of 238 adjacent normal samples at ≥5% frequency.

### Target sequencing

We expanded our investigation to include an additional 23 non-serous EOC tumors and five granulosa cell tumors with sequencing data covering 1,321 genes selected based on their suspected involvement in cancer, independently of the data obtained during exome sequencing. They were selected through a review of the literature, mutation databases and key pathways in tumorigenesis such as growth factor signaling, DNA damage response, p53 signaling, cell cycle control and apoptosis^35^ (Table 2, Supplementary Table 2). Approximately 1.4 GB of targeted gene sequencing data per sample was obtained by sequencing 1,321 genes covering 3.8 Mb (Supplementary Table 5). Mutations were cross referenced with COSMIC to identify driver mutations (Fig. 1C, Supplementary Table 6).

**Table 2:** .Samples used in target gene sequencing.

Case	Histology	Age at diagnosis
TG1	Endometrioid carcinoma, squamous differentiation	NA
TG2	Endometrioid adenocarcinoma	51
TG3	Endometrioid adenocarcinoma	54
TG4	Endometrioid adenocarcinoma	71
TG5	Endometrioid adenocarcinoma	60
TG6	Endometrioid adenocarcinoma	56
TG7	Endometrioid adenocarcinoma	61
TG8	Endometrioid adenocarcinoma	58
TG9	Endometrioid adenocarcinoma	54
TG10	Endometrioid adenocarcinoma	50
TG11	Endometrioid adenocarcinoma	61
TG12	Endometrioid adenocarcinoma	52
TG13	Endometrioid adenocarcinoma	51
TG14	Endometrioid adenocarcinoma	79
TG15	Granulosa Cell	NA
TG16	Granulosa Cell	NA
TG17	Granulosa Cell	61
TG18	Granulosa Cell	73
TG19	Granulosa Cell	66
TG20	Mucinous cystadenocarcinoma	78
TG21	Mucinous cystadenocarcinoma	30
TG22	Mucinous, carcinoma	55
TG23	Mucinous, carcinoma	51
TG24	Clear cell, carcinoma	62
TG25	Clear cell, carcinoma	64
TG26	Clear cell, carcinoma	53
TG27	Clear cell adenocarcinoma	63
TG28	Clear cell, carcinoma	63

We found 11 unique *TP53* mutations, seen in 10/28 (36%) tumors. *TP53* mutations were prevalent in endometrioid (6/14) and mucinous (3/4) subtypes but less frequent in clear cell (1/5) and absent in granulosa cell (0/5). We also observed the presence of *FOXL2* p.C134W mutation in 4/5 (80%) of the additional granulosa cell samples. Although other *FOXL2* mutations were observed in different samples, the p.C134W driver mutation was only observed in the granulosa cell subtype. *KRAS* mutations were observed in 3/4 (75%) of additional mucinous samples and 3/14 (21%) of additional endometrioid samples. *ARID1A* and *CTNNB1* were mutated more frequently in endometrioid tumors (6/14, 43% and 7/14, 50% respectively), further suggesting these are important contributors to that tumor type^31^. *ARID1A* truncating mutations were also recurrently observed in 2/5 (40%) clear cell tumors.

### Comparison to TCGA serous ovarian and serous/non-serous endometrial cancers

**Figure 2:**
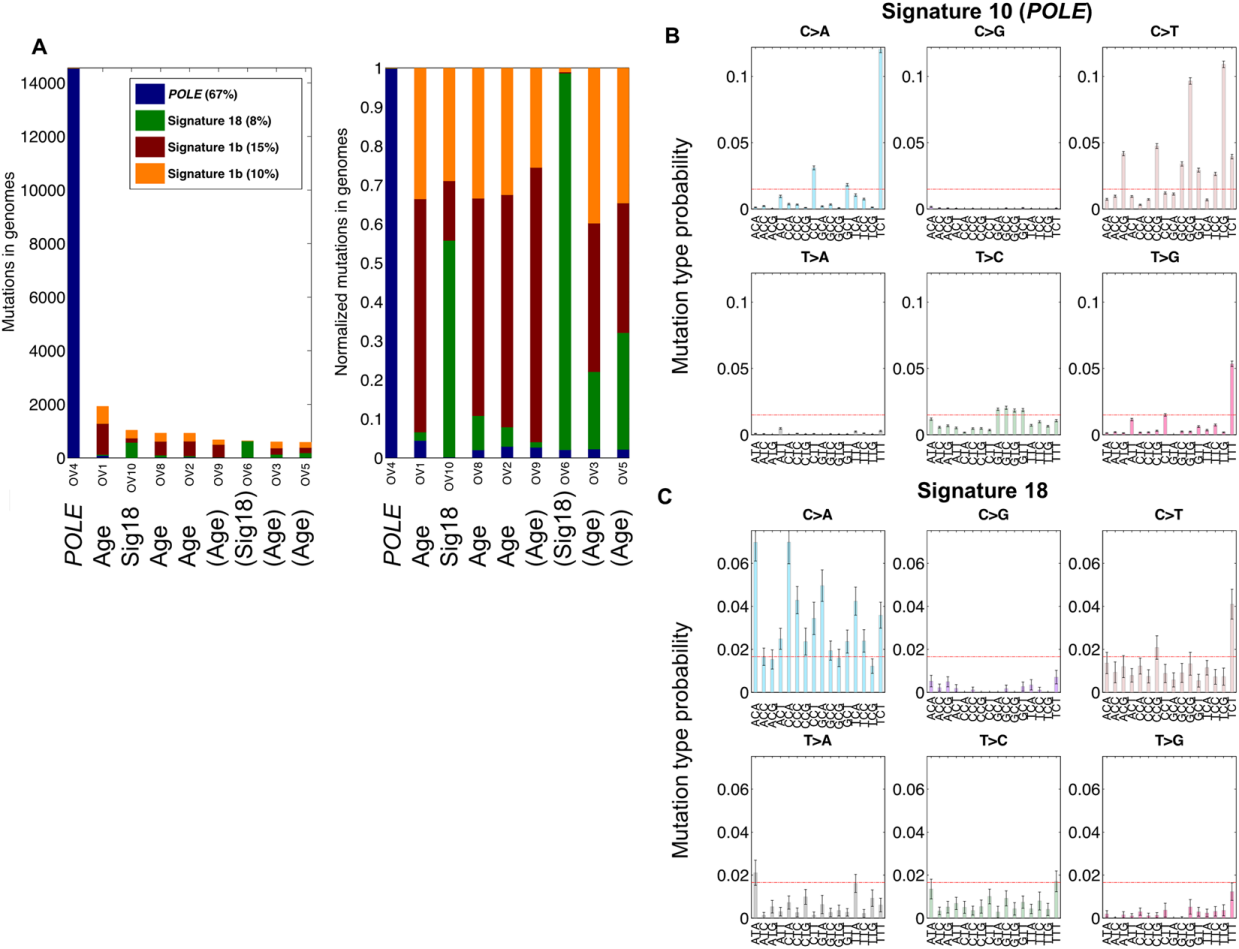
Mutation signatures of non-serous ovarian tumors. (**A**) Incidence of most probable mutation signatures in each sample by raw mutation count (left) or mutation fraction (right). The inferred mutation signature is listed below sample names. Parentheses indicate lower confidence in the assigned signature due to lower mutation counts. (**B**) Mutation distribution similar to Signature 10 (*POLE* proofreading domain dysfunction) from^38^. (**C**) Mutation distribution similar to Signature 18 from^38^.

We compared mutation patterns observed in our set of 9 whole exome ovarian tumors and our set of 23 targeted gene ovarian tumors to TCGA studies on serous ovarian^8^ and endometrial cancers^36^ at genes known to be mutated in these diseases (Fig. 1D). Serous ovarian cancer is marked by very high mutation rate of *TP53* (96%) and of few other cancer genes. Serous endometrial also has a high *TP53* mutation rate of 74%, as well as *PIK3CA* (36%), *FBXW7* (26%), and *PPP2R1A* (23%). *TP53* mutation rates were much lower in our non-serous OC samples: 10% in ES, and 36% in our TGS cohort. *PIK3CA* mutation rates were, on average, higher in our non-serous OC samples compared to serous cancers. We observed higher mutation rates in each of the following genes in non-serous ovarian samples compared to serous ovarian and serous endometrial: *PTEN*, *PIK3R1*, *CTNNB1*, *ARID1A*, and *KRAS*. Many of these genes are frequently mutated in non-ultramutated endometrioid uterine cancer (Fig. 2), particularly *PTEN*, *PIK3CA*, *PIK3R1*, *CTNNB1*, *ARID1A*, and *KRAS* ^36^.

### POLE mutations

One endometrioid sample (OV4) was observed to have very high mutation count (Fig. 1) (Supplementary Table 3). Further investigation revealed that this sample contained a *POLE* p.V411L mutation in the proofreading domain that has been previously observed in endometrioid uterine carcinoma^36^ and colorectal cancer^37^. Two additional *POLE* mutations were observed in endometrioid ovarian samples in our expanded TGS cohort: p.P286R (TG3) and another p.V411L (TG10).

### Mutation signatures

**Figure 3:**
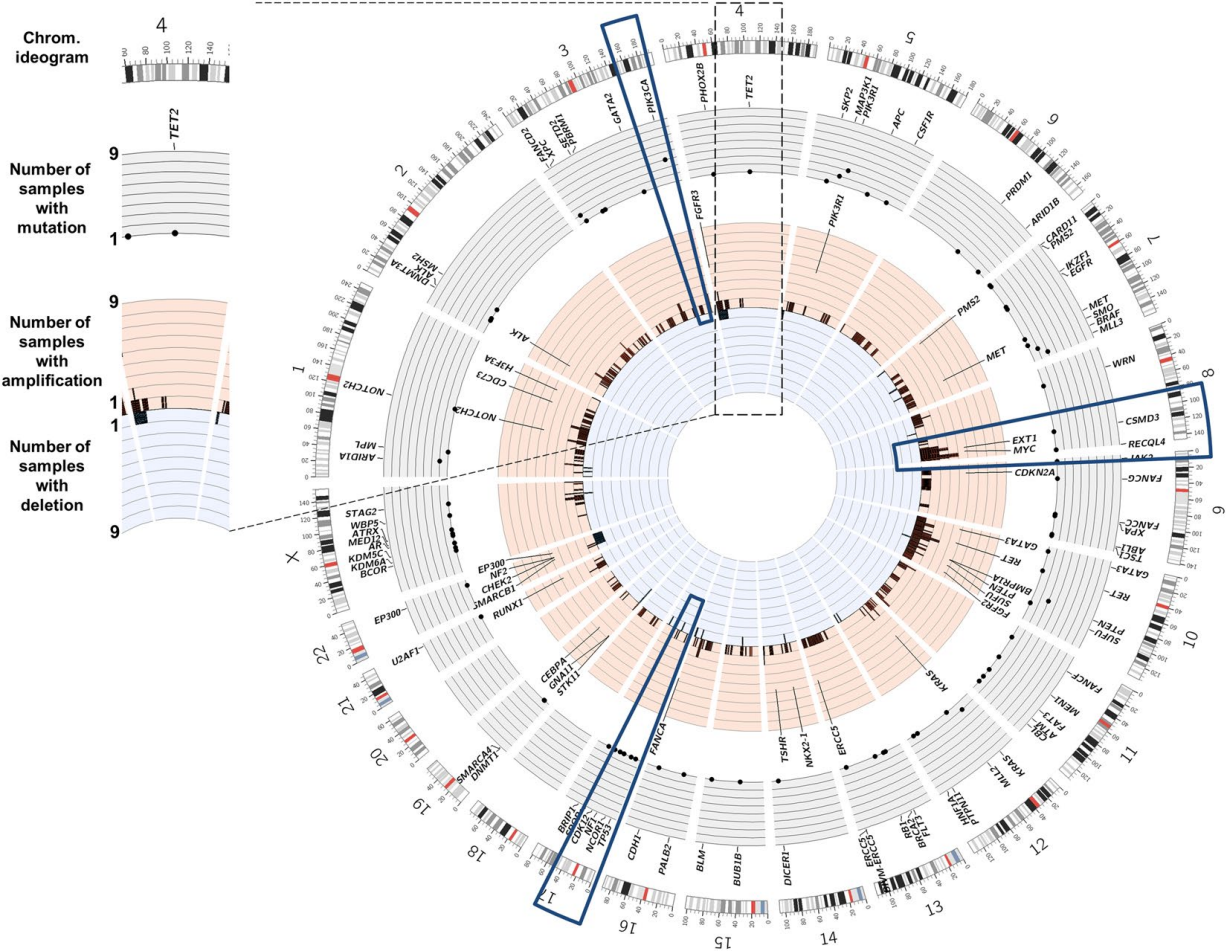
Mutational landscape across non-serous ovarian tumors. Left: (**A**) Circos plot display detected somatic alterations in Exome Sequencing (ES) tumors. Right: From outmost to inner most ring, chromosome ideogram, number of samples with mutations (light grey, increasing towards outside), number of samples with amplification (light red, increasing towards outside), numbers of samples with deletion (light blue, increasing towards inside). Dark blue slices highlight recurrent features: counterclockwise from top-left: *PIK3CA* mutations, amplification in *MYC* region, lack of mutations or deletions in *TP53* region.

Examining the pattern of nucleotide changes, *i.e*. the mutational signatures, in a tumor can suggest mechanisms that may have contributed to tumorigenesis. All three endometrioid ovarian tumors containing *POLE* proofreading domain mutations consisted almost entirely of the previously reported *POLE* mutation signature (TCT > TAT, TCG > TTG, TTT > TGT; Signature 10) (http://cancer.sanger.ac.uk/cosmic/signatures)^38^ (Fig. 2A,B). This signature was visible in the tumor-only analysis despite the lower somatic mutation specificity we have previously observed (Teer *et al*. unpublished observation). In ES samples, we observed signature 1b “Age” (likely age-related cytosine-deamination) in all samples and signature 18, which has unknown etiology, as the predominant signature in two samples of different histologies (OV10: mucinous and OV6: endometrioid) (Fig. 3A,C).

### Copy number alterations

Tumor specific copy number alterations (CNA) were examined in the ES dataset. A median of 25 events was observed in each sample (range 5–192) covering a median of 76,303,334 bases (range 1,818,739–661,026,741) (Supplementary Table 7). Although many events were observed in OV7, we suspected these were unreliable due to the very low coverage in the sample from normal plasma, and did not consider this sample further. As many of the alterations were large (median = 236,356 bases, range 1,291–47,327,598 bases), genes that have been previously described as cancer drivers^39^ were identified in the CNA regions.

Between 0 and 13 driver genes fell in copy number altered regions in each sample (Supplementary Table 8). Driver genes in amplified segments included *FGFR3*, *MYC*, *KRAS*, *EXT1*, and *PTEN*. Genes in deleted segments included *STK11*, *NF2*, *CHEK2*, *PMS2*, *CDKN2A*, and *FANCA* (Fig. 3). No sample contained a copy number alteration overlapping *TP53*, *BRCA1* or *BRCA2* (Fig. 3, Supplementary Table 8). Recent TCGA genomic classifications of high-grade serous ovarian adenocarcinoma and endometrial carcinomas revealed high levels of somatic CNAs in high grade serous ovarian and serous endometrial tumors, including recurrent amplifications in *MYC*, and *CCNE1*. We observed fewer CNAs in non-serous ovarian tumors, including only 2 samples with a *MYC* amplification and no samples with a *CCNE1* amplification.

Interestingly, sample OV3, an endometrioid tumor from the youngest individual in our cohort (diagnosed at 28 years of age) had the most CNAs. This tumor had the third lowest number of mutations with no identified common cancer mutations (Supplementary Table 4; Fig. 1). Altered driver genes in OV3 were mainly amplified, and included *NOTCH2*, *ALK*, *PIK3R1*, and *MET*. Two tumor suppressor genes were also amplified: *PTEN* and *CDKN2A*. Only one deletion was observed, which contained *PMS2*.

## Discussion

Here, we performed exome sequencing of ten incident cases of non-serous stage 1 ovarian neoplasms to determine somatic mutations and copy number alterations. We also performed target gene sequencing for 1,321 genes in 28 non-serous tumors to explore the mutation landscape of non-serous ovarian tumors. A limitation of our ES cohort study is a relatively small sample size and reported frequencies should be taken with caution. Despite this limitation, the availability of normal matched DNA allowed an improved identification of relevant variants. We also used an additional independent cohort. The data integration and comparison with publically available data led to five main conclusions.

First, non-serous ovarian tumors seem to be distinct from serous subtypes in their prevalence of *TP53* pathogenic variants. While previous studies have reported mutations in 96% of high grade serous epithelial tumors^8^, only one ES sample (OV8; mucinous) had a *TP53* mutation and no samples had deletions in the region. We examined the fraction of bases with depth of coverage ≥ 10 across the *TP53* gene, and found an average of 73.7% per sample, suggesting our coverage was sufficient to have observed mutations. In the TGS cohort, mutations were prevalent in mucinous (4/7, 57%), endometrioid (6/20, 30%) and clear cell (1/5, 20%) subtypes but still markedly lower than high grade serous. This is line with previous studies of clear cell carcinomas reporting 10–15% *TP53* mutation frequency^9, 40^. Interestingly, comparative studies of *TP53* mutations in uterine cancer also reports widely different mutation frequencies between the endometrioid (11.4–17%) and serous carcinomas (>90%)^36, 41^.

Second, a small but significant fraction of endometrioid tumors display mutations in the proofreading domain of *POLE*, and mutations are not found in other non-serous subtypes. One ES sample (OV4; endometrioid) harbored a *POLE* mutation in the proofreading domain, and showed a mutational signature matching a previously reported signature of *POLE* deficiency^38^. In the TGS set we observed recurrent *POLE* mutations in 14% (2/14) of endometrioid ovarian cancers. A screen for *POLE* mutations in a study of 251 Chinese samples of different subtypes of ovarian carcinomas, identified mutations in 3 out of 37 (8.1%) patients with ovarian endometrioid carcinoma, but not observed in the other subtypes^42^. Interestingly, *POLE* hotspot mutations were found in 7% (17/248) of uterine carcinomas, all of endometrioid histology^36^.

Third, we confirmed the high prevalence (5/6; 83%) of pathognomonic mutations in *FOXL2* in adult granulosa cell tumors. This is consistent with existing reports showing a prevalence ranging from 70.4% to 100%^43–50^.

Fourth, different non-serous subtypes have distinct mutation profiles. *KRAS* mutations are observed in significant fraction of mucinous and, to a lesser extent, in endometrioid but much less frequently (0.6%; 2/316) in high grade serous ovarian tumors^8^. A total of 71% (5/7) of mucinous samples carried mutations in *KRAS*, confirming its high frequency in this subtype^51–53^. We also observed 20% (4/20) of endometrioid tumors with mutations in *KRAS*. Mutations in *PIK3CA* and *ARID1A* are the most frequent alteration in clear cell carcinomas found in combination in 2/5 (40%) of all CCC tumors in the present study. Sequencing of 97 OCCC tumors identified a high frequency of mutations in *PIK3CA* (33%) and in *TP53* (15%)^9^.

Finally, the finding that deletion in genes implicated in the DNA damage response are relatively common suggest that therapeutic approaches targeting these pathways, such as the use of PARP inhibitors may be provide effective options in treatment. In summary, our results show that non-serous ovarian tumors are mutationally distinct from serous ovarian tumors. Interestingly, a similar but not identical mutational profile can be found in endometrioid ovarian and endometrioid uterine tumors. These similarities support the link between endometrioid ovarian cancers and endometriosis^31^. Our findings also offer molecular evidence to support continued distinction of non-serous ovarian cancers by histology, and may offer the opportunity for future work to investigate subtype-specific interventions targeting unique molecular alterations.

## Materials and Methods

### Participants

For the Exome Sequencing (ES) cohort all subjects were women between the ages of 28 and 63 (median 51) from Moffitt Cancer Center. Tumor and matched normal samples were retrieved from Moffitt’s Total Cancer Care tissue bank. Tumor samples for sequencing were selected to have >80% tumor cellularity. Six endometrioid, three mucinous, and one granulosa cell ovarian tumors were sequenced (Table 1). The study was approved by the Institutional Review board of the University of South Florida and all methods were performed in accordance with the relevant guidelines and regulations.

For the Target Gene Sequencing (TGS) cohort all samples were from the Total Cancer Care tissue bank. Patients were women between the ages of 30 and 79 (median 60) diagnosed in 1993–2012. Tumors underwent macrodissection to achieve >80% tumor cellularity in subsequent molecular studies. Samples were grouped by subtype: fourteen endometrioid, four mucinous, five clear cell, and five granulosa cell tumors were target sequenced (Table 2). All patients in both cohorts have provided informed consent under the Total Cancer Care protocol.

### Pathology review

We conducted a pathology review of all 10 cases used in exome sequencing. Details of the findings are presented in Table 1. Pathology reviews performed on hematoxylin and eosin (H&E) stained sections from a formalin-fixed paraffin-embedded (FFPE) block made during tissue acquisition at Moffitt’s Tissue Bank. Samples for sequencing were derived from the same dissected tumor samples used for FFPE and pathology review. For samples OV2, OV6, OV8, and OV9, original H&E sections were not available and new cuts were made from the original corresponding FFPE blocks and stained. In these cases it is possible that the section may not represent the local tumor section used for sequencing. Representative images are shown in Supplementary Figure 3.

### DNA isolation

DNAs from snap frozen ovarian tumor and from normal uterus were isolated using Qiagen DNeasy Blood & Tissue Kit (Qiagen). DNA from blood was isolated using Qiagen Gentra Puregene Blood Kit or Qiagen PAXgene blood DNA kit.

### Exome Sequencing

Paired-end libraries were constructed and whole exome capture was performed using Roche NimbleGen SeqCap EZ v3 (Roche). Captured libraries were sequenced using 100 base-pair reads on an Illumina HiScanSQ platform (Illumina, Inc., San Diego, CA) following the manufacturer’s protocols. Reads were aligned to the human genome reference hs37d5 using the Burrows-Wheeler Aligner (BWA)^54^. The Genome Analysis ToolKit (GATK)^55^ was used for insertion/deletion realignment, and quality score recalibration.

Somatic mutations were detected with either Strelka^56^ or MuTect^57^. Strelka 1.0.13 was run with default settings except the following: ssnvNoise = 0.00000005, sindelNoise = 0.0000001. MuTect 1.1.4 was run with default settings except: max_alt_alleles_in_normal_count = 3, max_alt_allele_in_normal_fraction = 0.05. ANNOVAR^58^ was used to annotate variants. Final read depth averaged 39.2×, and a median of ~87% of bases across samples had ≥10 reads. Coverage in each sample across genes of interest can be seen in Supplementary Figure 2. Copy number alterations were detected using EXCAVATOR^59^. Circos was used for visualization of somatic alterations^60^. Blood was unavailable for case OV7, and although plasma was subjected to exome capture, sequencing coverage was low (0.2% target bases having ≥10 reads) and duplicate rates were high (87%) preventing sensitive tumor/normal mutation detection. Case OV7 was analyzed with GATK, and mutations were enriched by filtering out known variants.

We also examined normal DNA to identify germline mutations implicated in ovarian cancer in breast/ovarian cancer families or in Lynch syndrome^30, 61–63^: *ATM, BARD1, BRCA1*, *BRCA2*, *BRIP1, CDH1, CHEK2, MLH1, MRE11A, MSH2, MSH6, MUTYH, NBN, PALB2, PMS1*, *PMS2, PTEN, RAD50*, *RAD51C*, *RAD51D, STK11*, and *TP53*.

### Targeted Gene Sequencing

Target gene capture was performed using SureSelect custom designs (Agilent Technologies, Inc., Santa Clara, CA) targeting 1,321 genes (Supplementary Table 2). Sequencing was performed using an Illumina GAIIx sequencing platform (Illumina, Inc., San Diego, CA) at the Beijing Genomics Institute (BGI, Shenzhen, China).

Sequences were aligned to the human genome reference hs37d5 using the Burrows-Wheeler Aligner (BWA)^54^. The Genome Analysis ToolKit (GATK)^55^ was used for insertion/deletion realignment, quality score recalibration, and variant identification. ANNOVAR^58^ was used to annotate mutations. Matched normal samples were not available for comparison so somatic mutations were enriched via population filtering, including 1000 Genomes and 238 unmatched normal samples. Final read depth averaged ~140x across the targeted regions and a median of ~94% of bases across samples had ≥10 reads (Supplementary Figure 2).

### TCGA mutation frequencies

Mutation frequencies were downloaded from cBioportal^64, 65^ using the “Ovarian Serous Cystadenocarcinoma^8^ and “Uterine Corpus Endometrial Carcinoma^36^.

### Mutation signatures

Mutational signatures were derived from the ES samples using previously described methods^38^. Curated and updated ICGC (International Cancer Genomics Consortium) signatures can be found at http://cancer.sanger.ac.uk/cosmic/signatures.

## Electronic supplementary material

Supplementary Data

Supplementary Tables 2–11

## References

[1] Kobel M (2013). Biomarker-based ovarian carcinoma typing: a histologic investigation in the ovarian tumor tissue analysis consortium. Cancer Epidemiol Biomarkers Prev.

[2] Kobel M (2010). Tumor type and substage predict survival in stage I and II ovarian carcinoma: insights and implications. Gynecologic oncology.

[3] Prat J (2012). Ovarian carcinomas: five distinct diseases with different origins, genetic alterations, and clinicopathological features. Virchows Archiv: an international journal of pathology.

[4] Foulkes WD, Gore M, McCluggage WG (2016). Rare non-epithelial ovarian neoplasms: Pathology, genetics and treatment. Gynecologic oncology.

[5] Shih IM, Kurman RJ (2004). Ovarian tumorigenesis: a proposed model based on morphological and molecular genetic analysis. The American journal of pathology.

[6] Kurman RJ, Shih IM (2008). Pathogenesis of ovarian cancer: lessons from morphology and molecular biology and their clinical implications. International journal of gynecological.

[7] Kurman RJ, Shih IM (2010). The origin and pathogenesis of epithelial ovarian cancer: a proposed unifying theory. The American journal of surgical pathology.

[8] Cancer Genome Atlas Research, N. Integrated genomic analyses of ovarian carcinoma. *Nature* **474**, 609–615 (2011). 10.1038/nature10166 PMC3163504 21720365

[9] Kuo KT (2009). Frequent activating mutations of PIK3CA in ovarian clear cell carcinoma. The American journal of pathology.

[10] Bonome T (2005). Expression profiling of serous low malignant potential, low-grade, and high-grade tumors of the ovary. Cancer research.

[11] Bast RC, Hennessy B, Mills GB (2009). The biology of ovarian cancer: new opportunities for translation. Nature reviews. Cancer.

[12] Song H (2009). A genome-wide association study identifies a new ovarian cancer susceptibility locus on 9p22.2. Nature genetics.

[13] Shen H (2013). Epigenetic analysis leads to identification of HNF1B as a subtype-specific susceptibility gene for ovarian cancer. Nature communications.

[14] Permuth-Wey J (2013). Identification and molecular characterization of a new ovarian cancer susceptibility locus at 17q21.31. Nature communications.

[15] Bojesen SE (2013). Multiple independent variants at the TERT locus are associated with telomere length and risks of breast and ovarian cancer. Nature genetics.

[16] Pharoah, P. D. *et al*. GWAS meta-analysis and replication identifies three new susceptibility loci for ovarian cancer. *Nature genetics* **45**, 362–370 (2013). 10.1038/ng.2564 PMC3693183 23535730

[17] Bolton KL (2010). Common variants at 19p13 are associated with susceptibility to ovarian cancer. Nature genetics.

[18] Chen K (2014). Genome-wide association study identifies new susceptibility loci for epithelial ovarian cancer in Han Chinese women. Nature communications.

[19] Kuchenbaecker, K. B. *et al*. Identification of six new susceptibility loci for invasive epithelial ovarian cancer. *Nature genetics* **47** (2015). 10.1038/ng.3185 PMC4445140 25581431

[20] Goode EL (2010). A genome-wide association study identifies susceptibility loci for ovarian cancer at 2q31 and 8q24. Nature genetics.

[21] Phelan CM (2017). Identification of 12 new susceptibility loci for different histotypes of epithelial ovarian cancer. Nature genetics.

[22] Kar SP (2016). Genome-Wide Meta-Analyses of Breast, Ovarian, and Prostate Cancer Association Studies Identify Multiple New Susceptibility Loci Shared by at Least Two Cancer Types. Cancer discovery.

[23] Kanchi KL (2014). Integrated analysis of germline and somatic variants in ovarian cancer. Nature communications.

[24] Martins FC (2014). Combined image and genomic analysis of high-grade serous ovarian cancer reveals PTEN loss as a common driver event and prognostic classifier. Genome biology.

[25] Ahmed AA (2010). Driver mutations in TP53 are ubiquitous in high grade serous carcinoma of the ovary. The Journal of pathology.

[26] Chien J (2015). TP53 mutations, tetraploidy and homologous recombination repair defects in early stage high-grade serous ovarian cancer. Nucleic acids research.

[27] Kelemen LE (2015). Genome-wide significant risk associations for mucinous ovarian carcinoma. Nature genetics.

[28] Ryland GL (2015). Mutational landscape of mucinous ovarian carcinoma and its neoplastic precursors. Genome medicine.

[29] Madore J (2010). Characterization of the molecular differences between ovarian endometrioid carcinoma and ovarian serous carcinoma. The Journal of pathology.

[30] Walsh T (2011). Mutations in 12 genes for inherited ovarian, fallopian tube, and peritoneal carcinoma identified by massively parallel sequencing. Proceedings of the National Academy of Sciences of the United States of America.

[31] Wiegand KC (2010). ARID1A mutations in endometriosis-associated ovarian carcinomas. The New England journal of medicine.

[32] Jones S (2010). Frequent mutations of chromatin remodeling gene ARID1A in ovarian clear cell carcinoma. Science.

[33] McConechy MK (2014). Ovarian and endometrial endometrioid carcinomas have distinct CTNNB1 and PTEN mutation profiles. Modern pathology: an official journal of the United States and Canadian Academy of Pathology, Inc.

[34] Walsh T (2010). Detection of inherited mutations for breast and ovarian cancer using genomic capture and massively parallel sequencing. Proc. Natl. Acad. Sci. USA.

[35] Greenawalt, D. H. *et al*. exome sequencing to understand tumor progression and identify targeted therapies. *In: Proceedings of the 103rd Annual Meeting of the American Association for Cancer Research; 2012 Mar 31-Apr 4; Chicago, IL. Philadelphia (PA): AACR: Cancer Research* **72**, doi:1538-7445.AM2012-435 (2012).

[36] Cancer Genome Atlas Research, N. *et al*. Integrated genomic characterization of endometrial carcinoma. *Nature* **497**, 67–73 (2013). 10.1038/nature12113 PMC3704730 23636398

[37] Cancer Genome Atlas, N. Comprehensive molecular characterization of human colon and rectal cancer. *Nature* **487**, 330–337 (2012). 10.1038/nature11252 PMC3401966 22810696

[38] Alexandrov LB (2013). Signatures of mutational processes in human cancer. Nature.

[39] Vogelstein B (2013). Cancer genome landscapes. Science.

[40] Ho, E. S. *et al*. p53 mutation is infrequent in clear cell carcinoma of the ovary. *Gynecologic oncology* **80**, 189–193 (2001). 10.1006/gyno.2000.6025 11161858

[41] Lax SF, Kendall B, Tashiro H, Slebos RJ, Hedrick L (2000). The frequency of p53, K-ras mutations, and microsatellite instability differs in uterine endometrioid and serous carcinoma: evidence of distinct molecular genetic pathways. Cancer.

[42] Zou Y (2014). Frequent POLE1 p.S297F mutation in Chinese patients with ovarian endometrioid carcinoma. Mutation research.

[43] Alexiadis M (2011). Nuclear receptor profiling of ovarian granulosa cell tumors. Hormones & cancer.

[44] D’Angelo E (2011). Prognostic significance of FOXL2 mutation and mRNA expression in adult and juvenile granulosa cell tumors of the ovary. Modern pathology: an official journal of the United States and Canadian Academy of Pathology, Inc.

[45] Takahashi A (2013). The FOXL2 mutation (c.402C > G) in adult-type ovarian granulosa cell tumors of three Japanese patients: clinical report and review of the literature. The Tohoku journal of experimental medicine.

[46] Schrader KA (2009). The specificity of the FOXL2 c.402C > G somatic mutation: a survey of solid tumors. PloS one.

[47] Jamieson S (2010). The FOXL2 C134W mutation is characteristic of adult granulosa cell tumors of the ovary. Modern pathology: an official journal of the United States and Canadian Academy of Pathology, Inc.

[48] Kim MS, Hur SY, Yoo NJ, Lee SH (2010). Mutational analysis of FOXL2 codon 134 in granulosa cell tumour of ovary and other human cancers. The Journal of pathology.

[49] Al-Agha OM (2011). FOXL2 is a sensitive and specific marker for sex cord-stromal tumors of the ovary. The American journal of surgical pathology.

[50] Gershon R (2011). FOXL2 C402G mutation detection using MALDI-TOF-MS in DNA extracted from Israeli granulosa cell tumors. Gynecologic oncology.

[51] Cuatrecasas M, Villanueva A, Matias-Guiu X, Prat J (1997). K-ras mutations in mucinous ovarian tumors: a clinicopathologic and molecular study of 95 cases. Cancer.

[52] Gemignani ML (2003). Role of KRAS and BRAF gene mutations in mucinous ovarian carcinoma. Gynecologic oncology.

[53] Vereczkey I (2011). Molecular characterization of 103 ovarian serous and mucinous tumors. Pathology oncology research.

[54] Li H, Durbin R (2009). Fast and accurate short read alignment with Burrows-Wheeler transform. Bioinformatics.

[55] DePristo MA (2011). A framework for variation discovery and genotyping using next-generation DNA sequencing data. Nature genetics.

[56] Saunders CT (2012). Strelka: accurate somatic small-variant calling from sequenced tumor-normal sample pairs. Bioinformatics.

[57] Cibulskis K (2013). Sensitive detection of somatic point mutations in impure and heterogeneous cancer samples. Nature biotechnology.

[58] Wang K, Li M, Hakonarson H (2010). ANNOVAR: functional annotation of genetic variants from high-throughput sequencing data. Nucleic acids research.

[59] Magi A (2013). EXCAVATOR: detecting copy number variants from whole-exome sequencing data. Genome biology.

[60] Krzywinski M (2009). Circos: an information aesthetic for comparative genomics. Genome research.

[61] Miki Y (1994). A strong candidate for the breast and ovarian cancer susceptibility gene BRCA1. Science.

[62] Boyd J, Rubin SC (1997). Hereditary ovarian cancer: molecular genetics and clinical implications. Gynecologic oncology.

[63] Lynch HT (1991). Hereditary nonpolyposis colorectal cancer (Lynch syndromes I & II). Genetics, pathology, natural history, and cancer control, Part I. Cancer genetics and cytogenetics.

[64] Gao J (2013). Integrative analysis of complex cancer genomics and clinical profiles using the cBioPortal. Science signaling.

[65] Cerami, E. *et al*. The cBio cancer genomics portal: an open platform for exploring multidimensional cancer genomics data. *Cancer discovery* **2**, 401-404.CD-12-0095 (2012). 10.1158/2159-8290.CD-12-0095 PMC3956037 22588877

