# Supplementary material for: Mutational heterogeneity in non-serous ovarian cancers
Source: Sci Rep. 2017 Aug 29;7:9728. doi: 10.1038/s41598-017-10432-9 (PMC5574976; doi:10.1038/s41598-017-10432-9)
Supplement: Supplementary file 1 — Supplementary Data [file 41598_2017_10432_MOESM1_ESM.pdf]

Supplementary Data for

Mutational heterogeneity in non-serous ovarian cancers

Jamie K. Teer[1], Sean Yoder[2], Anxhela Gjyshi[3,4], Santo V. Nicosia[5], Chaomei Zhang[2], Alvaro N.A. Monteiro[3]

[1]Department of Biostatistics and Bioinformatics, [2]Molecular Genomics Core Facility, and [3]Cancer Epidemiology Program, H. Lee Moffitt Cancer Center and Research Institute, Tampa, FL 33612, USA; [4]University of South Florida Cancer Biology PhD Program, Tampa, FL 33612, USA; [5]Department of Pathology and Cell Biology, USF Morsani School of Medicine, University of South Florida, Tampa, FL 33612, USA

Supplementary Figures and Tables Legends:

Included in this pdf file:

Supplementary Figure 1. REMARK-style diagram of study flow.

Supplementary Figure 2. Sequencing coverage distribution across samples. ***A.*** Exome sequencing samples. ***B.*** Target gene sequencing samples. Genes with 0 coverage were not targeted.

Supplementary Figure 3. . Representative histopathology of samples used in exome sequencing. Cases (see Table 1 for specific histologies and grades). A-B, OV1; C-D, OV2; E-F, OV3; G-H, OV4; I-J, OV5; K-L, OV6; M-N, OV7; O-P, OV8; Q-R, OV9; S-T, OV10. Original magnifications: left panels, x 63; right panels, x250.

Supplementary Table 1. Mutation landscape of ovarian tumors in published studies.

Included as a separate Excel file:

Supplementary Table 2. List of genes in target gene sequencing.

Supplementary Table 3. Exome sequencing mutation metrics.

Supplementary Table 4. Mutations in COSMIC's cancer genes in individual exome sequencing samples.

Supplementary Table 5. Target gene sequencing mutation metrics.

Supplementary Table 6. Mutations in COSMIC's cancer genes in individual target gene sequencing samples.

Supplementary Table 7. Metrics of Copy number alteration analysis of ES samples.

Supplementary Table 8. Cancer genes located in regions of copy number alterations in ES samples.

Supplementary Table 9. Germline variants found in exome sequencing in genes implicated in ovarian cancer susceptibility.

Supplementary Table 10. Annotation of notable germline variants found in individual samples.

Supplementary Table 11. List of all (filtered) somatic non-synonymous variants found the exome data.

Supplementary Fig. 1.

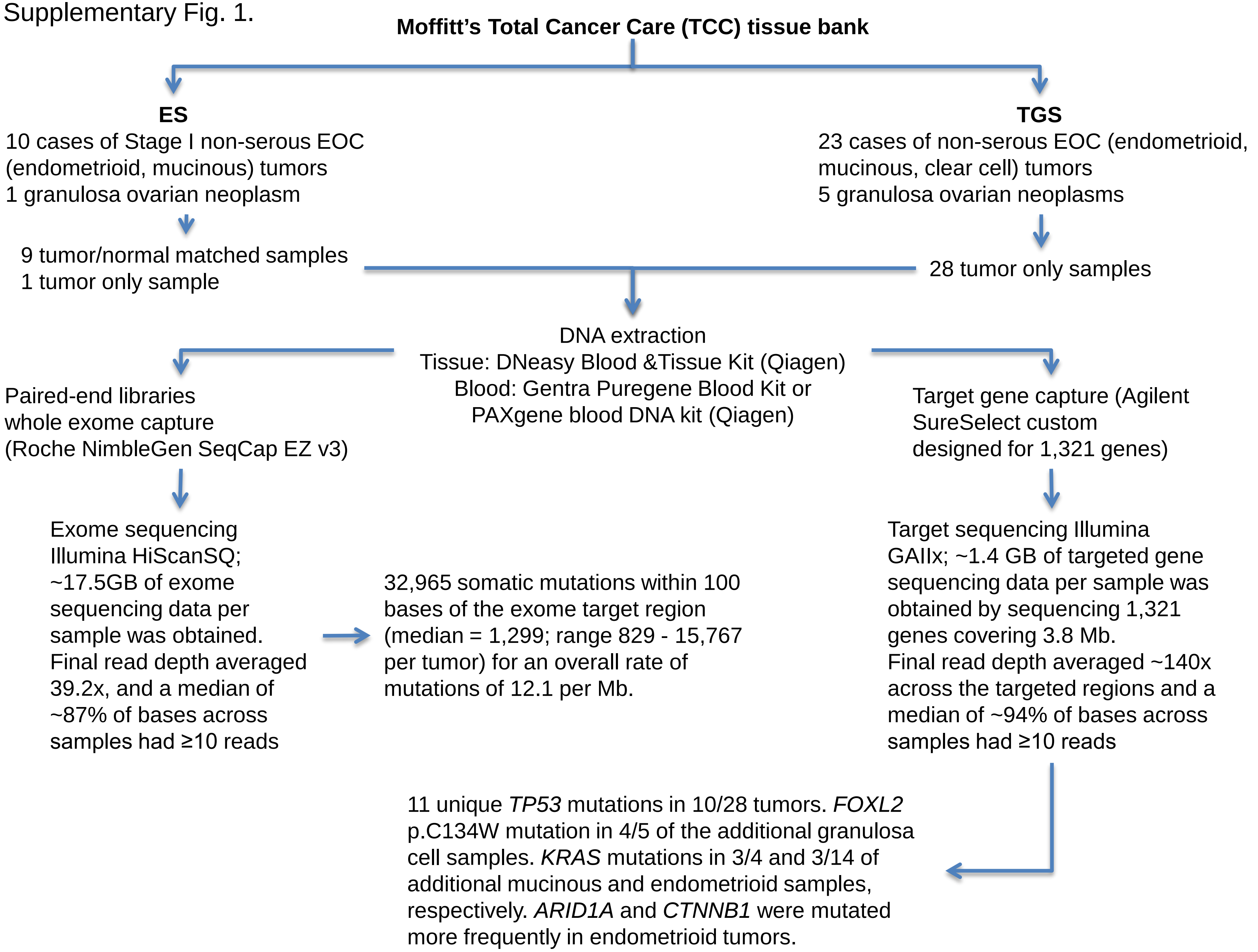

Supplementary Fig. 2.

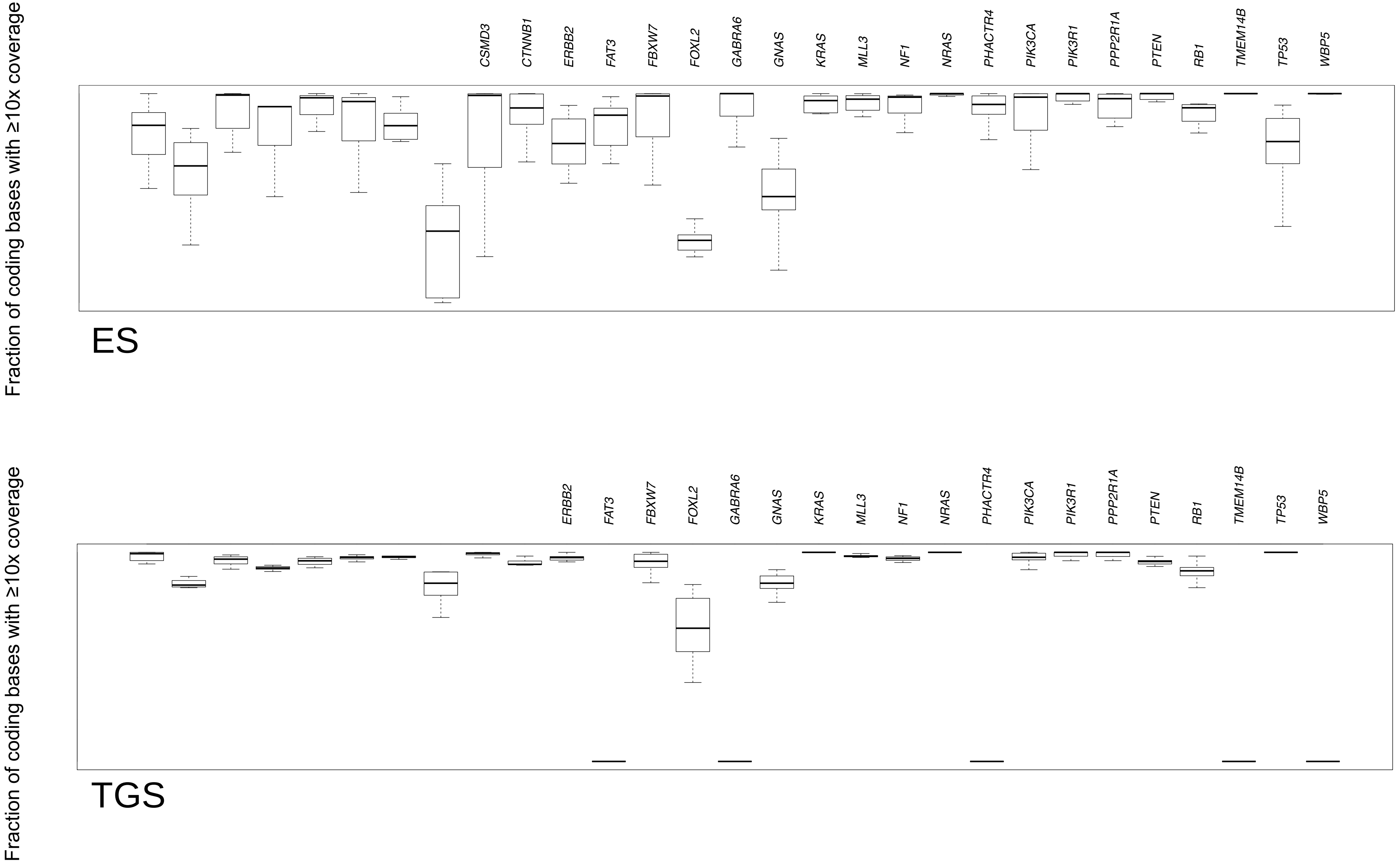

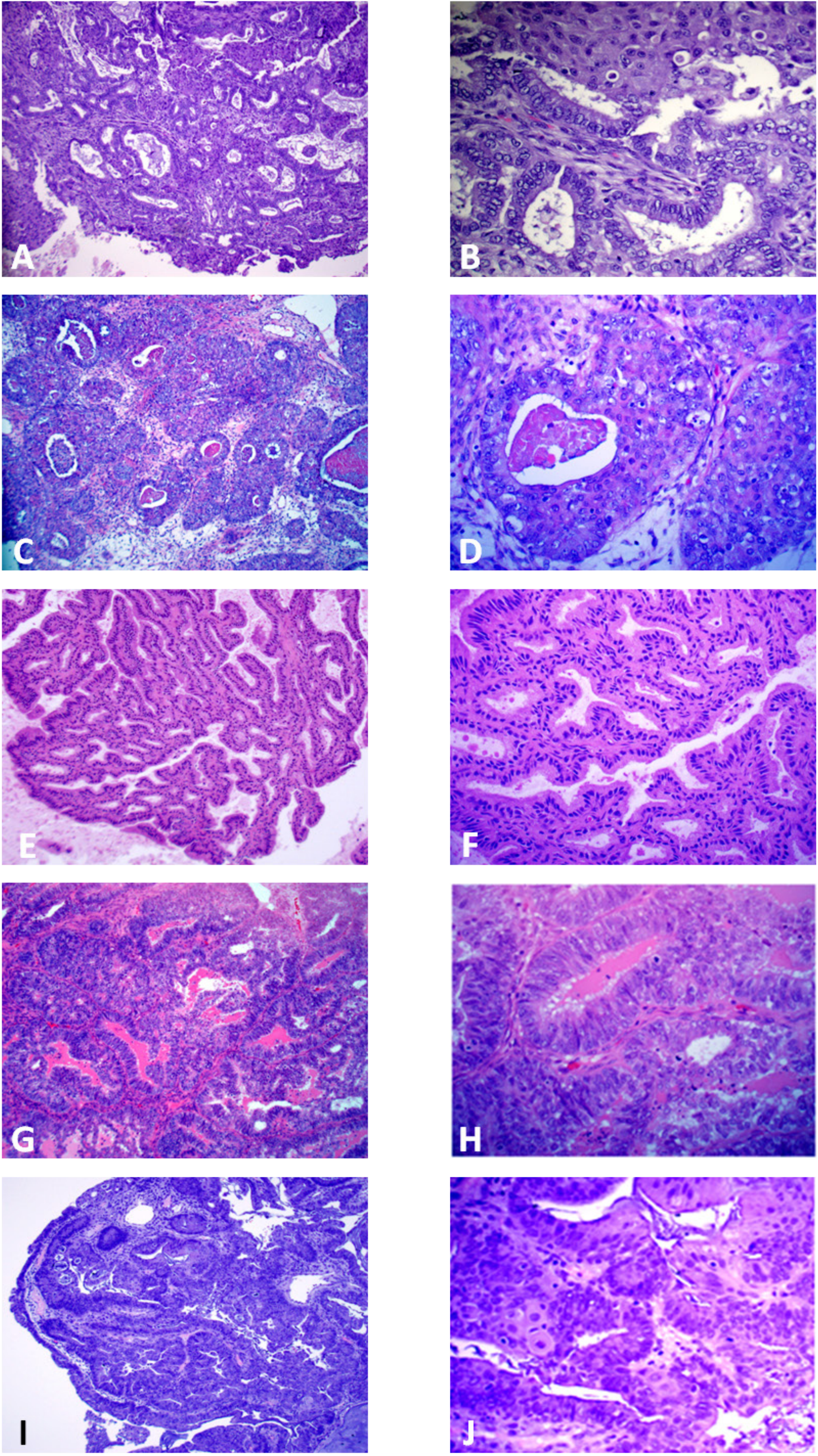
A
B
C
D
E
F
G
H
I
J

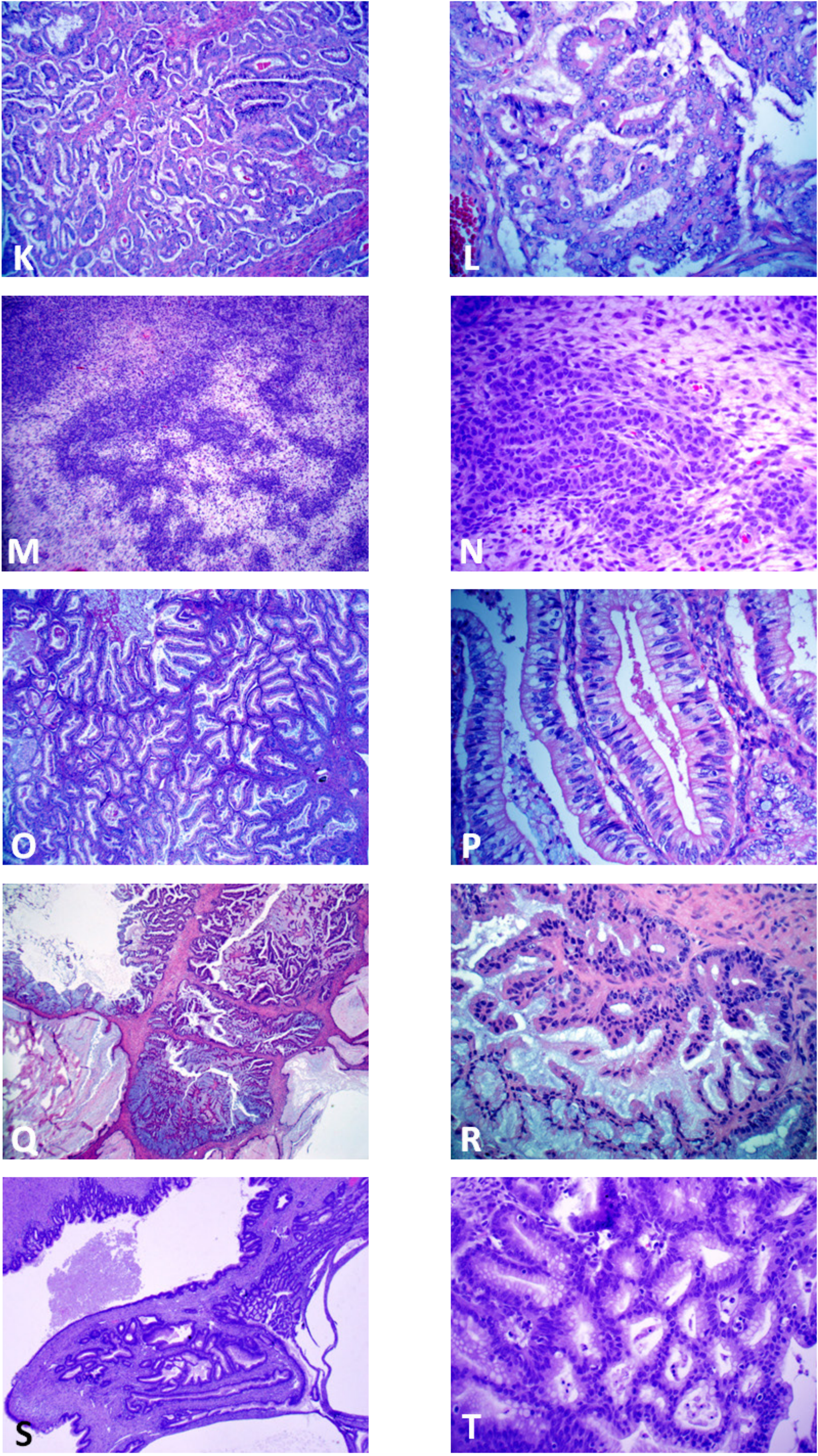
K
L
M
N
O
P
Q
R
S
T

Supplementary Table S1. Mutational landscape of epithelial ovarian carcinomas.

Study	Subtypes	Design and Methods	Summary and Highlights
(Gemignani et al., 2003)	104 primary invasive EOC - 21 serous, 30 endometrioid, 31 clear cell and 22 mucinous	Targeted Sanger sequencing (SS) for *KRAS* exon 2 and *BRAF* exons 11 and 15	Activating *KRAS* mutations were common in mucinous tumors (50%) but not in the other histologic types (5% in all of them combined). No mucinous tumor was found to harbor a *BRAF* mutation
(Kuo et al., 2009)	97 Ovarian Clear Cell Carcinoma (CCC)	Targeted SS for *KRAS*, *BRAF*, *PIK3CA*, *TP53*, *PTEN*, *CTNNB1*	*PIK3Ca* was the most frequently mutated gene, detected in 33% of the cases, followed by *TP53* in 15%, *KRAS* 7%, *PTEN* 5%, *CTNNB1* 3%, and *BRAF* in 1% of the cases.
(Jones et al., 2010)	8 CCC tumors, plus 34 additional CCC tumor/normal tissues for validation	Exome sequencing (ES), and SS for validation	4 genes: *PIK3CA, KRAS, PPP2R1A, ARID1A* were mutated in at least 2 of the 8 tumors studied. In total, after validation, mutations were identified for *PIK3CA* in 40% of the cases, *KRAS* 4.7%, *ARID1A* 57%, and *PPP2R1A* in 7.1% of the 42 tumors.
(Madore et al., 2010)	endometrioid and serous carcinomas	Gene expression analysis, TMA, Immunohisto-chemistry (IHC)	*TP53* mutation common among endometrioid carcinomas (EC) with a serous-like gene expression profile. Loss of *PTEN* protein expression observed in 30% (12/40) of EC and in 13% (12/111) of serous carcinomas. Strong association between loss of *PTEN* and overall survival was observed in serous EOC but not EC.
(Shih Ie et al., 2011)	209 ovarian and 56 uterine tumors of various histologic subtypes	Targeted massively parallel sequencing (MPS) for exons 5 and 6 of *PP2R1A*	*PPP2R1A* mutations were found in 10 of 110 (9.1%) of type I (low-grade) ovarian tumors of different histological subtypes. None of 71 type II ovarian (high-grade serous) carcinomas exhibited *PPP2R1A* mutations. In the uterine tumors, *PPP2R1A* mutations were observed in 2 of 30 type I uterine (endometrioid) carcinomas (6.7%) and 5 of 26 type II uterine (serous) carcinomas (19.2%).
(Cancer Genome Atlas Research, 2011)	489 high-grade serous (exome for 316)	High affinity hybrid exome capture and MPS	Characterized by *TP53* mutations (96% of tumors). *BRCA1/2* mutations found in 20% of the tumors. Other statistically recurrent mutated genes include: *RB1*, *NF1*, *FAT3*, *CSMD3*, *GABRA6*, and *CDK12*.
(Vereczkey et al., 2011)	80 serous tumors (borderline LG & HG), and 23 mucinous tumors(borderline and invasive)	PCR followed by melting point analysis for *BRAF* and *KRAS*. IHC for P53	The majority of mucinous ovarian tumor cases showed a *KRAS* mutation. No *BRAF* or *TP53* mutations were found in these cases

(Jones et al., 2012)	8 affinity-purified low-grade serous carcinomas (LGSC), plus 9 low grade and 10 serous borderline tumors for validation	Exome sequencing by MPS, validation with SS	Overall, *BRAF* and *KRAS* mutations occurred in 10 (38%) and 5 (19%) of 27 low-grade tumors, respectively. Among 15 morphologically pure LGSCs, four contained *KRAS* mutations, three contained *BRAF* mutations and one had a *PIK3CA* mutation.
(Ross et al., 2013)	48 relapsed OC cases-38 (80%) serous, 5 (10%) endometrioid, 3 (6%) clear cell, 1 mucinous (2%) and 1 (2%) undifferentiated carcinomas	Targeted MPS for 182 cancer-related genes	Most common genomic alterations were found in *TP53* (79%), *MYC* (25%), *BRCA1/2* (23%), *KRAS* (16.6%), and *NF1* (14.5%). *TP53* mutations were observed in 79% serous, 40% endometrioid and 33% CC carcinomas. All 8 (100%) of the ovarian tumors that featured *BRCA1* mutations were serous carcinomas. 3/5 (60%) of *ARID1A* mutations occurred in non-serous tumors.
(Tsang et al., 2013)	23 recurrent LGSC patients with a known initial diagnosis of ovarian serous borderline tumor (OSBT)	PCR, SS followed by deep sequencing of *KRAS*, *BRAF*	*KRAS* mutations are very common in recurrent LGSC, found in 10/23 (43.5%) patents, while *BRAF* mutations are rare, detected only in 1/23 (4.3%) patient. Deep sequencing detected low-abundance *KRAS* mutations in 8 additional patients (total 78.3%). *KRAS* G12V mutation was associated with shorter survival times.
(Takahashi et al., 2013)	4 adult-type granulosa cell tumors (AGCT) in Japanese patients	IHC & SS of *FOXL2*	Study identified 3/4 (75%) tumors harboring the c.402C>G mutation. Literature review revealed ~70.4-100% incidence of *FOXL2* c.402C>G (p.C134W) somatic mutation in AGCTs.
(Kanchi et al., 2014)	429 serous ovarian carcinoma cases and 557 controls	Germline and somatic exome sequencing	FA pathway genes were frequent targets of somatic and germ-line mutations in 20% of cases. Significantly mutated genes include previously reported *TP53*, *NF1*, *RB1*, *CDK12*, *BRCA1*, and new *SMG* and *KRAS*. Enrichment of rare truncations found in *BRCA1*, *BRCA2*, and *PALB2*. Germ-line truncation variants observed in genes not previously associated with ovarian cancer susceptibility (*NF1, MAP3K4, CDKN2B* and *MLL3*).
(McConechy et al., 2014)	33 cases of ovarian endometrioid & 307 cases of endometrial endometrioid carcinomas	Exon-capture for *ARID1A*, *PTEN*, *TP53*, *PIK3CA*, *KRAS*, *CTNNB1*, *PPP2R1A*. SS to verify *CTNNB1* mutation frequency	*PTEN* mutations were found in 67% of low-grade endometrial endometrioid carcinomas, but only in 17% of low-grade ovarian endometrioid carcinomas. *CTNNB1* mutations were identified in 53% of ovarian endometrioid carcinomas and in only 28% of endometrial endometrioid carcinomas. Mutation frequencies of *PIK3CA*, *ARID1A*, *PPP2R1A*, *KRAS*, and *TP53* are not significantly different.

(Zou et al., 2014)	251 Chinese samples-76 serous, 43 CCC, 37 ovarian endometrioid, 33 germ cell, 15 mucinous, 18 sex-cord stromal, and 25 other	direct sequencing of *POLE1*	A heterozygous somatic *POLE1* mutation, p.S297F (c.890C>T), but not p.P286R & p.V411L hotspot mutations observed in other cancer types, was identified in 3 out of 37 (8.1%) patients with ovarian endometrioid carcinoma. This mutation coexisted with mutation in the ovarian cancer-associated *PPP2R1A*.No *POLE1* mutations were identified in patients with other subtypes of ovarian carcinoma.
(Hoang et al., 2015)	89 ovarian endometrioid carcinomas	Targeted sequencing of *POLE* exonuclease domain, validated by SS	*POLE* mutations found in 4 of 89 (4.5%) cases. All were somatic missense point mutations, occurring at the commonly reported hotspots in endometrial carcinoma, P286R and V411L. Compared to findings in endometrium, *POLE* mutations in ovarian endometrioid carcinoma occur at much lower frequency and are observed only in low-grade tumors
(Ryland et al., 2015)	86 ovarian carcinomas including HG serous & endometrioid, LG endometrioid, clear cell & mucinous subtypes	Sequencing of target-enriched DNA libraries for 980 genes in regions of LOH	Significantly mutated genes include *TP53*, *PTEN* and *CDKN2A*. A high proportion of somatic truncating mutations found in TP53 (25/58; 43%) and *BRCA1* (2/2; 100%), compared to an overall truncating mutation frequency of 13 % (72/561). For 53 cases with *TP53* or *BRCA1* mutations that had SNP data available, 50 (94%) showed LOH of the wild-type allele, in contrast to other candidate genes 181/520 (35%). Genes with more than 50% deleterious mutations included *PTEN*, *CDKN2A*, *MAP2K4*, *PIK3R1*, *RB1*, *FANCA* and *BRCA1*.
(Rechsteiner et al., 2013)	142 formalin-fixed, paraffin-embedded EOC, including serous (n=63), endometrioid (n= 29), clear cell (n=25), mu cinous (n=14), and others (n=11)	pyro-sequencing targeting mutations in TP53 exons 5-8, KRAS exons 2 and 3, and BRAF exon 15	*TP53* mutations occurred frequently not only in high-grade serous carcinomas (58.7%), but also in mucinous (57%) and clear cell EOC (52%). *KRAS* mutations were mainly identified in mucinous EOC (57%) and were found concomitantly with *TP53* mutations in five mucinous carcinomas (36%). *BRAF* mutations seem to be a rare event in EOC.
(Mackenzie et al., 2015)	69 mucinous ovarian tumors	Targeted massively parallel sequencing (MPS) for 50 genes commonly mutated in human cancer	Mutations were detected in *KRAS*, *TP53*, *CDKN2A*, *PIK3CA*, *PTEN*, *BRAF*, *FGFR2*, *STK11*, *CTNNB1*, *SRC*, *SMAD4*, *GNA11* and *ERBB2*. *KRAS* mutations were the most frequently observed alteration (64.9%). *TP53* mutations occurred in 56.8% of mucinous carcinomas. RAS-pathway activating changes were observed in all but one tumor. Concurrent *ERBB2* amplification and *KRAS* mutation were observed in 11% of cases (7/63).

References

Cancer Genome Atlas Research, N. (2011). Integrated genomic analyses of ovarian carcinoma. Nature *474*, 609-615.

Gemignani, M.L., Schlaerth, A.C., Bogomolniy, F., Barakat, R.R., Lin, O., Soslow, R., Venkatraman, E., and Boyd, J. (2003). Role of KRAS and BRAF gene mutations in mucinous ovarian carcinoma. Gynecologic oncology *90*, 378-381.

Hoang, L.N., McConechy, M.K., Kobel, M., Anglesio, M., Senz, J., Maassen, M., Kommoss, S., Meng, B., Postovit, L., Kelemen, L.E.*, et al.* (2015). Polymerase Epsilon Exonuclease Domain Mutations in Ovarian Endometrioid Carcinoma. International journal of gynecological cancer : official journal of the International Gynecological Cancer Society *25*, 1187-1193.

Jones, S., Wang, T.L., Kurman, R.J., Nakayama, K., Velculescu, V.E., Vogelstein, B., Kinzler, K.W., Papadopoulos, N., and Shih Ie, M. (2012). Low-grade serous carcinomas of the ovary contain very few point mutations. The Journal of pathology *226*, 413-420.

Jones, S., Wang, T.L., Shih Ie, M., Mao, T.L., Nakayama, K., Roden, R., Glas, R., Slamon, D., Diaz, L.A., Jr., Vogelstein, B.*, et al.* (2010). Frequent mutations of chromatin remodeling gene ARID1A in ovarian clear cell carcinoma. Science *330*, 228-231.

Kanchi, K.L., Johnson, K.J., Lu, C., McLellan, M.D., Leiserson, M.D., Wendl, M.C., Zhang, Q., Koboldt, D.C., Xie, M., Kandoth, C.*, et al.* (2014). Integrated analysis of germline and somatic variants in ovarian cancer. Nature communications *5*, 3156.

Kuo, K.T., Mao, T.L., Jones, S., Veras, E., Ayhan, A., Wang, T.L., Glas, R., Slamon, D., Velculescu, V.E., Kuman, R.J.*, et al.* (2009). Frequent activating mutations of PIK3CA in ovarian clear cell carcinoma. The American journal of pathology *174*, 1597-1601.

Mackenzie, R., Kommoss, S., Winterhoff, B.J., Kipp, B.R., Garcia, J.J., Voss, J., Halling, K., Karnezis, A., Senz, J., Yang, W.*, et al.* (2015). Targeted deep sequencing of mucinous ovarian tumors reveals multiple overlapping RAS-pathway activating mutations in borderline and cancerous neoplasms. BMC Cancer *15*, 415.

Madore, J., Ren, F., Filali-Mouhim, A., Sanchez, L., Kobel, M., Tonin, P.N., Huntsman, D., Provencher, D.M., and Mes-Masson, A.M. (2010). Characterization of the molecular differences between ovarian endometrioid carcinoma and ovarian serous carcinoma. The Journal of pathology *220*, 392-400.

McConechy, M.K., Ding, J., Senz, J., Yang, W., Melnyk, N., Tone, A.A., Prentice, L.M., Wiegand, K.C., McAlpine, J.N., Shah, S.P.*, et al.* (2014). Ovarian and endometrial endometrioid carcinomas have distinct CTNNB1 and PTEN mutation profiles. Modern pathology : an official journal of the United States and Canadian Academy of Pathology, Inc *27*, 128-134.

Rechsteiner, M., Zimmermann, A.K., Wild, P.J., Caduff, R., von Teichman, A., Fink, D., Moch, H., and Noske, A. (2013). TP53 mutations are common in all subtypes of epithelial ovarian cancer and occur concomitantly with KRAS mutations in the mucinous type. Experimental and molecular pathology *95*, 235-241.

Ross, J.S., Ali, S.M., Wang, K., Palmer, G., Yelensky, R., Lipson, D., Miller, V.A., Zajchowski, D., Shawver, L.K., and Stephens, P.J. (2013). Comprehensive genomic profiling of epithelial ovarian cancer by next generation sequencing-based diagnostic assay reveals new routes to targeted therapies. Gynecologic oncology.

Ryland, G.L., Doyle, M.A., Goode, D., Boyle, S.E., Choong, D.Y., Rowley, S.M., Li, J., Australian Ovarian Cancer Study, G., Bowtell, D.D., Tothill, R.W., *et al.* (2015). Loss of heterozygosity: what is it good for? BMC medical genomics *8*, 45.

Shih Ie, M., Panuganti, P.K., Kuo, K.T., Mao, T.L., Kuhn, E., Jones, S., Velculescu, V.E., Kurman, R.J., and Wang, T.L. (2011). Somatic mutations of PPP2R1A in ovarian and uterine carcinomas. The American journal of pathology *178*, 1442-1447.

Takahashi, A., Kimura, F., Yamanaka, A., Takebayashi, A., Kita, N., Takahashi, K., and Murakami, T. (2013). The FOXL2 mutation (c.402C>G) in adult-type ovarian granulosa cell tumors of three Japanese patients: clinical report and review of the literature. The Tohoku journal of experimental medicine *231*, 243-250.

Tsang, Y.T., Deavers, M.T., Sun, C.C., Kwan, S.Y., Kuo, E., Malpica, A., Mok, S.C., Gershenson, D.M., and Wong, K.K. (2013). KRAS (but not BRAF) mutations in ovarian serous borderline tumour are associated with recurrent low-grade serous carcinoma. The Journal of pathology *231*, 449-456.

Vereczkey, I., Serester, O., Dobos, J., Gallai, M., Szakacs, O., Szentirmay, Z., and Toth, E. (2011). Molecular characterization of 103 ovarian serous and mucinous tumors. Pathology oncology research : POR *17*, 551-559.

Zou, Y., Liu, F.Y., Liu, H., Wang, F., Li, W., Huang, M.Z., Huang, Y., Yuan, X.Q., Xu, X.Y., Huang, O.P., *et al.* (2014). Frequent POLE1 p.S297F mutation in Chinese patients with ovarian endometrioid carcinoma. Mutation research *761*, 49-52.
